# ^13^C-Metabolic Flux Analysis Reveals Effect of Phenol on Central Carbon Metabolism in *Escherichia coli*

**DOI:** 10.3389/fmicb.2019.01010

**Published:** 2019-05-07

**Authors:** Sayaka Kitamura, Yoshihiro Toya, Hiroshi Shimizu

**Affiliations:** Department of Bioinformatic Engineering, Graduate School of Information Science and Technology, Osaka University, Osaka, Japan

**Keywords:** phenol toxicity, *Escherichia coli*, ^13^C-metabolic flux analysis, enzymatic assay, citrate synthase, acetate overflow metabolism

## Abstract

Phenol is an important chemical product that can be used in a wide variety of applications, and it is currently produced from fossil resources. Fermentation production of phenol from renewable biomass resources by microorganisms is highly desirable for sustainable development. However, phenol toxicity hampers phenol production in industrial microorganisms such as *Escherichia coli*. In the present study, it was revealed that culturing *E. coli* in the presence of phenol not only decreased growth rate, but also biomass yield. This suggests that phenol affects the carbon flow of the metabolism, but the mechanism is unknown. To investigate the effect of phenol on the flux distribution of central carbon metabolism, ^13^C-metabolic flux analysis (^13^C-MFA) was performed on cells grown under different phenol concentrations (0, 0.1, and 0.15%). ^13^C-MFA revealed that the TCA cycle flux reduced by 25% increased acetate production from acetyl-CoA by 30% in the presence of 0.1% phenol. This trend of flux changes was emphasized at a phenol concentration of 0.15%. Although the expression level of citrate synthase, which catalyzes the first reaction of the TCA cycle, does not change regardless of phenol concentrations, the *in vitro* enzyme activity assay shows that the reaction was inhibited by phenol. These results suggest that the TCA cycle flux decreased due to phenol inhibition of citrate synthase; therefore, ATP could not be sufficiently produced by respiration, and growth rate decreased. Furthermore, since carbon was lost as acetate due to overflow metabolism, the biomass yield became low in the presence of phenol.

## Introduction

Phenol is a valuable compound used in various applications, such as in the production of polycarbonate, epoxy resin, phenol resin, and aniline (Schmidt, 2005). Although it is mainly produced by the cumene method using benzene derived from fossil resources at present (Wierckx et al., 2005), fermentation production using microorganisms from renewable biomass resources is required to achieve sustainable development and solve environmental problems. A tyrosine phenol-lyase (TPL), which has been discovered in some microorganisms such as *Bacterium coli phenologenes*, *Pantoea agglomerans*, and *Clostridium tetanomorphum*, catalyzes the conversion between tyrosine and phenol (Brot et al., 1965; Kumagai et al., 1970). Microbial phenol production has been reported in *Pseudomonas putida* by overexpressing the heterologous TPL of *P. agglomerans* (Wierckx et al., 2005). More recently, engineered strains of *Escherichia coli*, widely used as industrial bio-production hosts, can produce phenol from glucose by introducing the TPL from *P. agglomerans* (Kim et al., 2014; Thompson et al., 2016). However, productivity is still low for industrial applications.

Organic solvents, including phenol, are toxic toward a wide range of microorganisms, including *E. coli*, and reduce their growth (Ramos et al., 2002). In the phenol-producing *E. coli* strain, there is a challenge of low phenol productivity due to toxicity (Kim et al., 2014). Phenol tolerance varies among microorganisms, with some species including *P. putida*, used for bioremediation, being able to tolerate the presence of phenol (Ramos et al., 2002). In the same gram-negative bacteria, however, *E. coli* is weak against phenol and stops growing under a 1.2 g/L, corresponding to 0.11%(v/v), phenol condition (Kim et al., 2014). Despite this disadvantage, *E. coli* is an excellent host for industrial applications because of its rapid growth rate, easy genetic manipulation, and abundant biological knowledge. Elucidation of the reasons why *E. coli* is weak against phenol would make possible to overcome this weakness and enhance phenol production.

Various organic solvents are toxic to *E. coli*, and this toxicity correlates with a logarithm of the partition coefficient in *n*-octanol and water (log P_ow_) (Aono et al., 1994). The log P_ow_ of phenol is 1.46 (Verschueren, 1983), and is very severe among organic solvents. In *E. coli*, some efflux pumps such as AcrAB-TolC and AcrEF-TolC are involved in solvent tolerance (Ramos et al., 2002). Furthermore, it has been reported that the composition of the cell membrane changes in the presence of phenol (Keweloh et al., 1990), and phenol tolerance increases by alterations of the fatty acid composition of membranes (Keweloh et al., 1991). Because its respiratory activity is not reduced in the presence of phenol (Keweloh et al., 1990), the cause of growth reduction would not be respiratory chain inhibition due to membrane damage. We found in this work that culturing *E. coli* in the presence of phenol not only decreased growth rate, but also biomass yield. This suggests that phenol affects the carbon flow of central carbon metabolism, but the mechanism is unknown. ^13^C-metabolic flux analysis (^13^C-MFA) is an effective approach to investigate the carbon flux distribution on central carbon metabolism (Wittmann, 2007; Zamboni et al., 2009).

In the present study, we cultured wild type *E. coli* under different phenol concentrations (0, 0.1, and 0.15%), and compared these flux distributions to identify the effect of phenol on the metabolism. To investigate the cause of the flux changes, the *in vitro* enzyme assay was performed and revealed that citrate synthase is strongly inhibited by phenol.

## Materials and Methods

### Strains and Culture Conditions

The *E. coli* strains used in this study are shown in Supplementary Table S1. In preculture, wild type, *gltA*+, and Δ*pta* strains were aerobically grown at 37°C overnight using 5 mL of M9 medium containing 4 g/L glucose in a test tube. For the *gltA*+ strain, 40 mg/mL histidine and 10 mg/mL Thiamine hydrochloride was supplemented to the M9 media in accordance with the previous report (Kitagawa et al., 2005).

To evaluate phenol toxicity using an automated optical density monitoring system, the preculture was inoculated into 5 mL of the same M9 medium in an L-shaped test tube at an initial optical density at 660 nm (OD_660_) of 0.05. Phenol was added to be 0, 0.04, 0.08, 0.1, 0.12, and 0.16% (v/v). The cultures were incubated at 37°C at 70 rpm using a TVS062CA incubator (Advantec, Tokyo, Japan). The OD_660_ was measured every 5 min.

For evaluating the fermentation profiles in flask scale, the preculture of wild type and Δ*pta* strains were inoculated into 50 mL of M9 medium containing 4 g/L glucose as the sole carbon source in a 200 mL baffled flask at an initial OD_600_ of 0.05, and incubated at 37°C at 200 rpm using a BR-43FL incubator (TAITEC, Saitama, Japan). Phenol was added to be 0 and 0.15% (v/v). For ^13^C-MFA, the cultures were performed using the same condition except that the glucose was replaced to [1-^13^C] glucose. Phenol was added to be 0, 0.1, and 0.15% (v/v). All cultures were performed in triplicate.

### Measurement of Cell Concentration and Extracellular Metabolites

The OD_600_ was measured using an UVmini-1240 UV-VIS spectrophotometer (Shimadzu, Kyoto, Japan). The dry cell weight (DCW) was calculated using a conversion coefficient of 0.3 g/L/OD_600_ based on a previous report (Soini et al., 2008). Concentrations of glucose, acetate, formate, ethanol, lactate, and succinate in the culture were measured using a high-performance liquid chromatography system (Shimadzu, Kyoto, Japan) with an Aminex HPX-87H column (Bio-Rad, Hercules, CA, United States). The detailed method was described in Okahashi et al. (2017). The detection limits were 5 mM for ethanol and 1 mM for organic acids including lactate, acetate, formate, and succinate.

### Measurement of Proteinogenic Amino Acids

During the mid-log phase, an appropriate amount of cells (0.0015 gDCW) was collected by centrifugation. After hydrolyzation, the proteinogenic amino acids were derivatized with tert-butyldimethylsilyl (tBDMS). The mass isotopomer distributions of amino acids were measured using a gas chromatography/mass spectrometer (GC/MS) (Agilent Technologies, United States) with a DB-5MS column (Agilent Technologies, United States). The mass isotopomer distributions were corrected for the presence of naturally occurring isotopes (van Winden et al., 2002). The detailed protocols for sample preparation and measurement were described in Okahashi et al. (2017).

### ^13^C-Metabolic Flux Analysis

The central carbon metabolism including glycolysis, the pentose phosphate pathway, the TCA cycle, the Entner–Doudoroff (ED) pathway, the anaplerotic pathway, and the glyoxylate shunt, was considered for ^13^C-MFA based on previous analyses of *E. coli*. The elementary metabolites unit framework was used for modeling the carbon atom transitions (Antoniewicz et al., 2007). All reactions and carbon atom transitions are summarized in Supplementary Table S2. Flux distribution was optimized to minimize a residual sum of square (RSS) between calculated and measured amino acid mass isotopomer distributions. The fitness function was described below:

$$\begin{array}{cc} \text{Minimize RSS} & =\sum_{i=1}^{n}{\left(\frac{MID_{i}^{measured}-MID_{i}^{simulated}}{SD_{i}}\right)}^{2}\\ & +\sum_{j=1}^{n}{\left(\frac{r_{j}^{measured}-r_{j}^{simulated}}{SD_{j}}\right)}^{2} \end{array}$$

where MID_i_^measured^ and MID_i_^simulated^ are measured and simulated mass isotopomer distribution of *i*th amino acid, respectively. The standard deviation (SD) was set to 0.01 in GC/MS measurements (Wada et al., 2017). The r_j_^measured^ and r_j_^simulated^ are measured and simulated flux of *j*th reaction, respectively. The difference between the calculated and measured values of the mass isotopomer distribution was statistically evaluated by the χ^2^ test. The 95% confidence intervals of each flux were evaluated by the grid search method (Antoniewicz et al., 2006). All calculations were performed using the OpenMebius software (Kajihata et al., 2014) with MATLAB 2018a (MathWorks, Natick, MA, United States).

### Measurement of Enzyme Activities

To measure the citrate synthase activity of the *E. coli* cells cultured at the different phenol concentrations, the cells obtained from the 1 mL culture were suspended in 0.1 M of Tris–HCl pH 8.2, and were disrupted by sonication repeating on/off 10 times at out level at 50% using a UD-100 ultrasonic homogenizer (Tomy Seiko, Tokyo, Japan). After removing the debris by centrifugation (20,000 ×*g*, 4°C, 30 min), the crude extract was used for citrate synthase activity measurement. The protein concentration of the crude extract was measured using a nanoDrop 2000 spectrophotometer (Thermo Fisher Scientific, Waltham, MA, United States). Citrate synthase activity was measured by Ellmann’s method, that detects the thiol group of CoA by a colorimetric reaction using 5′,5′-Dithiobis(2-nitrobenzoic acid) (DTNB) (Srere, 1969). DTNB was measured at 412 nm by UVmini-1240 UV-VIS spectrophotometer (Shimadzu, Kyoto, Japan). After incubating a mixture containing 10 μL of crude extract, 190 μL of MilliQ water, 12.5 μL of 10 mM acetyl-CoA, and 25 μL of 1 mM DTNB in 0.1 M Tris–HCl (pH 8.2) at 25°C for 5 min, 12.5 μL of 10 mM oxaloacetate in 0.1 M Tris–HCl (pH 8.2) was added to start the reaction. The absorbance at 412 nm was measured every 10 s.

Phenol inhibition of the *E. coli* citrate synthase *in vitro* reaction was examined using a sample obtained from a *gltA* overexpressing strain, JW0710-AM, from the ASKA library (Kitagawa et al., 2005). In preculture, a single colony of this mutant was inoculated in 5 mL of Luria-Bertani (LB) medium, and was incubated overnight at 37°C at 150 rpm. The preculture was inoculated into 50 mL of LB medium at an initial OD_660_ of 0.05, and was incubated at 37°C at 150 rpm using a BR-43FL incubator (TAITEC, Saitama, Japan). At OD_600_ of approximately 0.3, 0.1 mM of isopropyl β-D-1-thiogalactopyranoside was added to induce gene expression. After 2 h, the cells were collected by centrifugation (3000 rpm, 4°C, 10 min), and were washed by 5 mL of filter sterilized buffer containing 50 mM sodium phosphate, 200 mM NaCl, and a protease inhibitor tablet (cOmplete tablets, Roche). To keep the plasmid overexpressing the *gltA* gene, 25 mg/L of chloramphenicol were added to the media. The obtained cell pellet was extracted by sonication and used for *in vitro* enzymatic assays in the same manner described above.

Phenol inhibition of glucose 6-phosphate isomerase (PGI) *in vitro* reaction was also evaluated using a sample obtained from wild type *E. coli*. PGI activity was measured as NADPH formation through a coupled reaction with glucose 6 phosphate dehydrogenase (G6PDH) (Salas et al., 1965; Peng and Shimizu, 2003). After addition of an appropriate amount of crude cell extract to a reaction mixture containing 0.1 M Tris–HCl (pH 7.8), 10 mM MgCl_2_, 0.5 mM NADP^+^, 1 U G6PDH (Sigma-Aldrich, Merck, Darmstadt, Germany), and 2 mM D-Fructose 6 phosphate, NADPH formation was measured by absorption at 340 nm. One unit (U) is defined as the amount of enzyme required to convert 1 μmol of substrate into product per minute per milligram protein.

## Results

### Evaluation of Phenol Toxicity in *E. coli*

**FIGURE 1:**
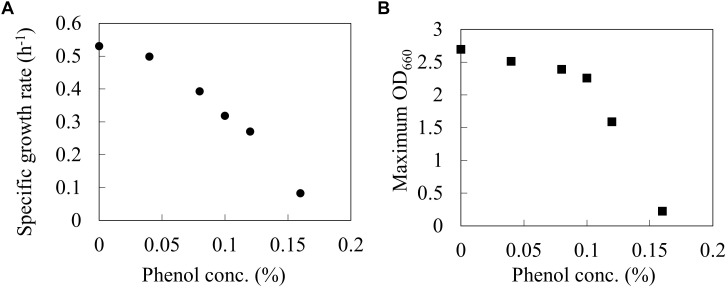
Relationships between phenol concentration and Specific growth rate **(A)**, and Maximum OD660 **(B)** on wild type *E. coli* BW25113. Closed circles and squares represent the maximum specific growth rate (h^-1^) and the maximum OD_660_ during the 24 h period.

The wild type *E. coli* BW25113 was cultured using a synthetic M9 medium with different phenol concentrations (0, 0.04, 0.08, 0.1, 0.12, and 0.16%) in L-shaped test tubes. Figure 1 shows the specific growth rate and the maximum OD_660_ at each phenol concentration. The specific growth rate decreased by 40% under 0.1% phenol, and by 84% under 0.16% phenol as compared to that without addition of phenol. A previous study has also reported that an increase in the phenol concentration of the culture led to a decrease in the growth rate of *E. coli*, and eventually cell growth was completely inhibited (Kim et al., 2014). These phenotypes were consistent with our result. Furthermore, the value of maximum OD_660_ also decreased by 16% under 0.1% phenol and by 92% under 0.16% phenol. The drastic decrease of biomass yield suggests that phenol affects the carbon flux of the central carbon metabolism.

### Growth Characteristics of *E. coli* Under Different Phenol Concentrations

**FIGURE 2:**
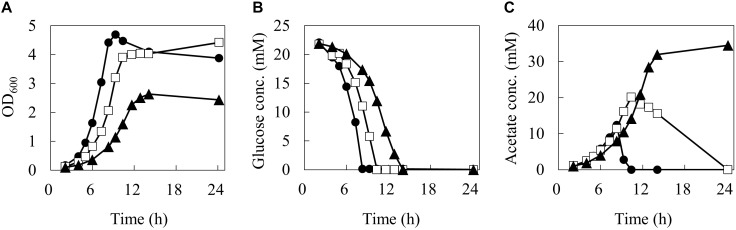
Batch growth profiles of wild type *E. coli* under different phenol concentrations. Time courses of biomass **(A)**, glucose **(B)**, and acetate **(C)** concentrations. Closed circles, open squares, and closed triangles represent measurement data series during 24 h by 0, 0.1, and 0.15% of phenol concentrations, respectively.

In order to reveal the influence of phenol on the carbon flow of the metabolism, the cells were cultured in 50 mL of M9 medium using a baffled flask with three different phenol concentrations (0, 0.1, and 0.15%). The time courses of cell, glucose, and acetate concentrations in the culture broth are shown in Figure 2.

At 0% phenol, the OD_600_ reached the maximum value of 4.7 at 9.3 h. Glucose consumption was the fastest among the three conditions, and was depleted by 8.3 h. Acetate concentration reached a maximum of 12.4 mM at 8.3 h, and was quickly consumed after glucose depletion. At 0.1% phenol, the maximum OD_600_ was 4.4 at 24.2 h. Glucose was consumed more slowly and was depleted by 10.4 h. Acetate concentration reached a maximum of 20.1 mM (1.6 times of that at 0% phenol) at 10.4 h, and then it was gently consumed. At 0.15% phenol, the maximum OD_600_ was significantly decreased to 2.6. Glucose consumption was the slowest and was depleted at 14 h. Acetate production was further enhanced to 34.5 mM at 24 h. No acetate consumption was observed after depletion of glucose. Furthermore, no ethanol, lactate, succinate, and formate productions were detected throughout the culture period under any conditions. The specific rates during the exponential growth phase are summarized in Table 1. Compared to the phenol 0% condition, the specific growth rate and specific glucose consumption rate at 0.15% phenol decreased by 42 and 24%, respectively, whereas the specific acetate production rate increased by 56%.

**Table 1:** Specific growth, glucose consumption, and acetate production rates of wild type *E. coli* under different phenol concentrations.

Phenol concentration %	Cell growth h^-1^	Glucose consumption mmol gDCW^-1^ h^-1^	Acetate production mmol gDCW^-1^ h^-1^
0.00	0.59 ± 0.002	9.43 ± 0.58	6.05 ± 0.16
0.10	0.46 ± 0.001	7.68 ± 0.17	7.31 ± 0.19
0.15	0.34 ± 0.001	7.10 ± 0.22	9.53 ± 0.23

**FIGURE 3:**
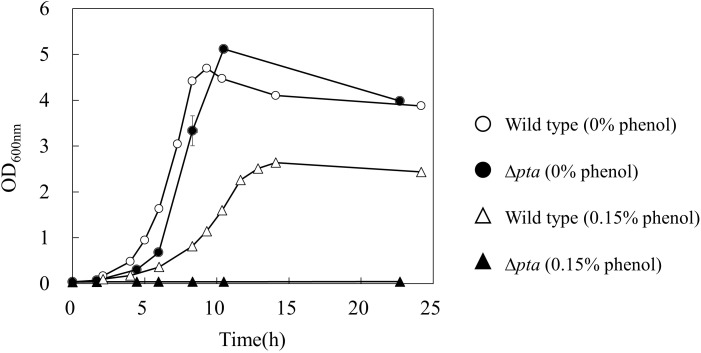
Effect of *pta* gene deletion on phenol tolerance in *E. coli*. The open and closed symbols represent the OD_600_ of wild type and Δ*pta* strains at 0% (circle) and 0.15% (triangle) phenol existence, respectively.

To further investigate the effect of acetate overflow on the phenol tolerance, a Δ*pta* strain which does not have phosphotransacetylase for acetate formation was cultured in the absence and presence of phenol (Figure 3). Interestingly, the growth profile of the Δ*pta* strain was similar to that of the wild type in the absence of phenol, whereas the strain did not grow in the presence of 0.15% phenol. The result suggests that the acetate overflow is related to *E. coli* growth in the presence of phenol.

### Flux Distributions Under Different Phenol Concentrations by ^13^C-Metabolic–Flux Analysis (^13^C-MFA)

To identify the reaction in which the flux was changed depending on phenol concentrations, a ^13^C-MFA was performed for each condition. The cells were cultured with [1-^13^C] glucose as a sole carbon source. The mass isotopomer distributions of proteinogenic amino acids at the exponential growth phase were measured by GC/MS. The mass isotopomer distributions of amino acids that have been corrected for natural isotope abundances are shown in Supplementary Table S3. The flux distributions were optimized to minimize the RSS between calculated and measured amino acid mass isotopomer distributions. The threshold of the χ^2^ test for the goodness of fit was 106.4 (104 independent data and 18 degrees of freedom for the model). As a result of flux optimization, the RSS values of conditions with 0, 0.1, and 0.15% phenol were 92.1, 104.5, and 103.3, respectively (passed χ^2^ test, *p* < 0.05). The 95% confidence interval of each flux, to explain the measured mass isotopomer distributions of amino acid, was also calculated. The best fitted flux distribution and the 95% confidence interval are shown in Supplementary Table S4.

**FIGURE 4:**
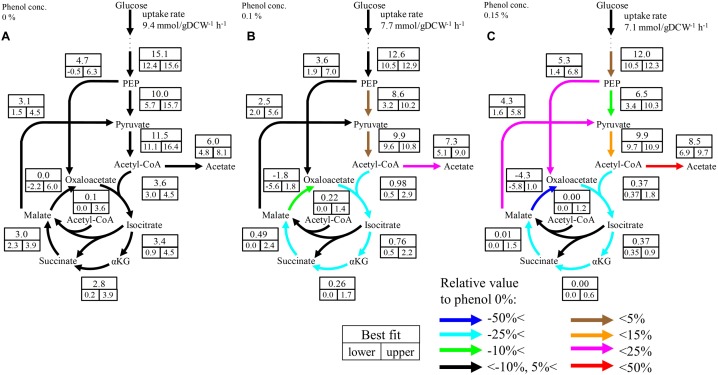
Flux distributions of the downstream pathways at 0% **(A)**, 0.1% **(B)**, and 0.15% **(C)** phenol existence. The unit of fluxes is shown as mmol gDCW^-1^ h^-1^. Values in the boxes: values in the upper boxes, and values in the lower boxes are best fit values and 95% confidence interval. Color of arrows indicates relative comparison of best fit values of metabolic fluxes to those in the 0% phenol condition. The detailed flux distributions of the overall pathways were described in Supplementary Table S4.

Although no significant change was observed in the upstream pathways including glycolysis, the pentose phosphate pathway, and the ED pathway by phenol addition, a large change occurred in the downstream pathways including pyruvate metabolism and the TCA cycle. The flux distributions focused on the downstream pathways are shown in Figure 4 The flux of the TCA cycle excluding malate dehydrogenase was significantly decreased in the presence of phenol. At the 0% phenol condition, the flux of citrate synthase reaction, the first reaction of the TCA cycle, was 3.6 mmol gDCW^-1^ h^-1^. However, the fluxes of citrate synthase at 0.1 and 0.15% phenol were decreased to 0.5 and 0.4 mmol gDCW^-1^ h^-1^, respectively. This suggests that acetyl-CoA does not flow into the TCA cycle, therefore, increasing overflow from acetyl-CoA to acetate.

Although the upper bound of the estimated flux of glyoxylate shunt seems to be decreased as the phenol concentration increases, it is difficult to conclude the flux changes because the confidence interval of this flux at the low phenol concentration. The flux of this pathway is usually low in *E. coli* under glucose carbon source without phenol addition (Leighty and Antoniewicz, 2012). Therefore, it is considered that this pathway did not work so much, regardless of the phenol concentration.

### Effect of Phenol on Enzyme Activities

^13^C-metabolic flux analysis revealed that the TCA cycle flux decreased in the presence of phenol in *E. coli*. Because no leakage of the TCA cycle intermediates, such as citrate and α-ketoglutarate, was detected in the culture broths, we assumed that phenol inhibits a citrate synthase reaction, at the entrance of the TCA cycle. In order to evaluate the changes in the protein expression level of citrate synthase, the specific activities of the crude extracts obtained from the cells cultured with three different phenol concentrations (0, 0.1, and 0.15%) were measured using an *in vitro* enzymatic assay. The specific activities of the crude cell extracts obtained from the cultures with 0, 0.1, and 0.15% phenol were 0.095 ± 0.007, 0.087 ± 0.009, and 0.100 ± 0.009 U, respectively. No significant difference was observed. This result suggests that the expression level of citrate synthase does not change among the culture conditions. Assuming 50% protein in the DCW, the citrate synthase activity (0.095 U) under the 0% phenol was calculated as 2.9 mmol/gDCW/h. In the ^13^C-MFA, the 95% confidence interval of this reaction was 3 to 4.5 mmol gDCW^-1^ h^-1^. Since the citrate synthase activity is almost consistent with the range of the flux, the *in vitro* enzyme assay results are reasonable.

**FIGURE 5:**
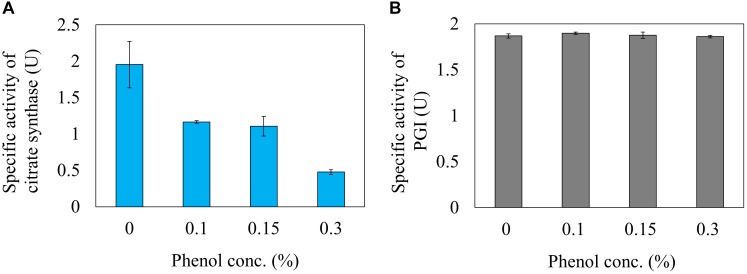
Inhibition of citrate synthase **(A)** and PGI **(B)** activities by the phenol *in vitro* reaction.

Next, the effect of phenol on the reaction rate of citrate synthase was investigated using a crude cell extract obtained from a *gltA* overexpressing strain. The relative activities of citrate synthase to a phenol free condition were 59.8, 56.6, and 24.6% at phenol concentrations in the reaction mixture of 0.1, 0.15, and 0.3%, respectively (Figure 5A). This result is consistent with the ^13^C-MFA results showing that the flux of the reaction from acetyl-CoA and oxaloacetate to citrate was reduced from 3.6 to 0.37 mmol gDCW^-1^ h^-1^ by addition of 0.15% phenol. Since high concentration of phenol is involved in protein denaturation, the decreased citrate synthase activity by phenol addition may be due to denaturation of the enzyme. To remove the suspicion, the effect of phenol on other enzyme activity was examined. As the representative enzyme, phenol inhibition of PGI was investigate (Figure 5B), because little flux change in the reaction was observed in the ^13^C metabolic flux analysis in response to phenol addition. No change in the PGI activity was observed by addition of phenol in the reaction mixture. This result supports that the cause of citrate synthase activity decrease in the presence of phenol is not denaturation of the protein.

If the inhibition of citrate synthase by phenol is the cause of growth reduction, the overexpression would restore the growth in the presence of phenol. To evaluate the effect of citrate synthase overexpression on phenol tolerance, the citrate synthase overexpressing (*gltA*+) strain was cultured using the M9 medium with different phenol concentrations. Because the expression of citrate synthase is regulated by an IPTG inducible T5-*lac* promoter (Kitagawa et al., 2005), 50 μM IPTG was added to the cultures. Supplementary Figure S1 shows the specific growth rate and the maximum OD_660_ at each phenol concentration. The decrease of cell growth was also observed in this overexpressing strain in the presence of phenol. Because the overexpression of citrate synthase caused a sever growth defect without phenol condition, fine-tuning of the expression level would be needed to alleviate the phenol stress.

## Discussion

In the present study, we investigated in detail the cause of phenol toxicity in *E. coli* from the viewpoint of metabolism. ^13^C-MFA showed an enhanced overflow of acetyl-CoA to acetate at phenol concentrations of 0.1 and 0.15%, indicating that the TCA cycle flux was significantly decreased. Further analysis based on measuring the specific enzyme activity *in vitro* revealed that citrate synthase, at the entrance reaction of the TCA cycle, was inhibited by phenol and led to the flux repression of this reaction.

^13^C-metabolic flux analysis can reveal how the flux changed on the metabolic pathways, but it does not provide the mechanism that caused this flux change. In addition, since it assumes a steady state condition, the flux change in a sequential pathway is found; however, it cannot identify which reaction in the pathway caused the flux change. The mechanism of flux changes must be discussed together with the knowledge about cellular systems, such as enzyme expression and reaction inhibition. For instance, the flux information alone cannot conclude whether the enhanced acetate production led to the decrease of the TCA cycle flux, or the decrease of the TCA cycle flux led to the enhanced acetate production. In *E. coli*, it is known that acetate synthesis occurs due to overflow metabolism (Castaño-Cerezo et al., 2009). Therefore, based on the citrate synthase activity assays *in vitro*, it is considered that the overflow to acetate increased because the TCA cycle flux was repressed by phenol.

How did the decrease of the TCA cycle flux occur in the presence of phenol? Because respiration and the TCA cycle are linked with NADH, damage to the respiratory chain may cause a decrease in the TCA cycle flux. If so, however, NADH will accumulate and lactate and ethanol will be produced in order to recycle NADH produced by glycolysis; like in anaerobic fermentation, these fermentation products were not detected in the culture broth analysis by HPLC. Furthermore, inhibition of the respiratory chain increases uptake of glucose in *E. coli* (Kihira et al., 2012), but the specific glucose uptake rate decreased in the presence of phenol (Table 1). If α-ketoglutarate dehydrogenase and aconitase are inhibited by phenol, α-ketoglutarate and citrate should overflow, respectively, but these compounds were not detected either by the culture broth analysis. Based on these findings, it is considered that the reaction stopped at the point of citrate synthase, which catalyzes the first step of the TCA cycle. In addition, the culture profiles in Figure 2 show that acetate was consumed promptly after depletion of glucose in the absence of phenol. On the other hand, consumption of acetate after glucose depletion was remarkably decreased at 0.1% phenol, and was completely stopped at 0.15% phenol. In *E. coli* grown with acetate as a carbon source, acetate is converted to acetyl-CoA and is catabolized via the glyoxylate shunt into the TCA cycle to avoid CO_2_ emission (Zhao and Shimizu, 2003). Therefore, the reaction in which phenol was directly inhibited is a common reaction for acetate catabolic pathways. The acetate uptake and the glyoxylate shunt do not work during the glucose consumption phase. Because reactions from acetyl-CoA to isocitrate are common, we focused on citrate synthase, which catalyzes this step.

Metabolic flux changes are caused by several factors, such as enzyme expression level, and reaction inhibition. Citrate synthase is an important enzyme that is regulated at the gene expression level by various transcription factors such as Cra, ArcAB, and Fnr (Matsuoka and Shimizu, 2011). First, in order to investigate the change in enzyme expression, the specific activity of the crude extract of the cells cultured at different phenol conditions was evaluated, but no activity change was observed. Next, in order to investigate whether phenol affected the citrate synthase reaction in *E. coli*, activity was evaluated by adding phenol to the *in vitro* reaction solution. As shown in Figure 5A, the enzyme activity decreased to 56.6% by addition of 0.15% phenol. The compound is highly permeable to the cell membrane due to its hydrophobicity. Assuming equal concentration of phenol inside and outside the cell, the examined phenol concentration affects the citrate synthase activity *in vivo*. This enzyme assay result suggests that phenol affects the enzyme reaction level, and not the gene expression level. In the ^13^C-MFA results, since the citrate synthase flux is more severely inhibited to 11% at 0.15% phenol, it suggests that other factors are remained to decrease the TCA cycle flux. A comprehensive analysis of gene expression profiles might provide clues to identify the factors.

For decreasing the acetate overflow in the presence of phenol, a gene knockout of *pta* encoding phosphotransacetylase which catalyzes the acetate formation pathway was investigated (Figure 3). The phenol tolerance of this mutant was lower than that of wild type strain. No growth of the Δ*pta* strain was observed in the presence of 0.15% phenol. Since the knockout mutant cannot discard the end product of glycolysis as acetate in the presence of phenol, excess intermediates would accumulate in the cell and inhibit the metabolic flow in glycolysis. As another hypothesis, post-translational modification of proteins by acetylation may have affected the metabolic behavior. Acetyl-phosphate, an intermediate in the acetate synthesis pathway, is an acetyl group donor for lysine acetylation of various proteins. It has been reported that the acetylation of isocitrate lyase affects the activity of glyoxylate shunt and transcription factor RcsB which related to an acid stress susceptibility (Castaño-Cerezo et al., 2014).

In the present study, ^13^C-MFA and enzyme assays reveal that the TCA cycle flux decreased due to phenol inhibition of citrate synthase; therefore, ATP could not be sufficiently produced by respiration, and growth rate decreased. Furthermore, since the carbon was lost as acetate due to overflow metabolism, the biomass yield became low in the presence of phenol. In the central carbon metabolism of *E. coli*, acetyl-CoA node is a branch point between the TCA cycle and acetate synthesis. The inhibition of citrate synthase by phenol blocks carbon flux into the TCA cycle and enhances the overflow into the acetate synthesis. Furthermore, since the acetyl-phosphate is an intermediate of acetate synthesis pathway, the accumulation may affect the TCA cycle flux through changes in enzyme activity and gene expression via protein acetylation. Investigations of the gene expression profiles and post-translational modifications on phenol stress would be necessary for further understanding the metabolic regulation. However, flux of upstream pathways such as glycolysis and the pentose phosphate pathway did not decrease. Phenol is synthesized via the aromatic amino acid synthesis pathway from phosphoenolpyruvate of glycolysis and erythrose 4-phosphate of the pentose phosphate pathway as precursors. So, although phenol has a negative effect on *E. coli* growth, phenol would not inhibit its own biosynthesis from glucose. Regarding phenol production by *E. coli*, then, it would be effective to separate the growth phase from the production phases, because the TCA cycle is not involved in phenol synthesis. The reaction for phenol synthesis should be suppressed during the growth phase, and should be induced after the cells are sufficiently grown.

## Author Contributions

SK, YT, and HS conceived the study. SK designed the study and performed all the experiments and computational analysis. YT designed the study and helped to do the experiments and computational analysis. HS designed and supervised the study. SK and YT drafted the manuscript. All authors contributed to preparing the final version of the manuscript and approved the manuscript to submit to this journal.

## Conflict of Interest Statement

The authors declare that the research was conducted in the absence of any commercial or financial relationships that could be construed as a potential conflict of interest.
