# Supplementary material for: 13C-Metabolic Flux Analysis Reveals Effect of Phenol on Central Carbon Metabolism in Escherichia coli
Source: Front Microbiol. 2019 May 7;10:1010. doi: 10.3389/fmicb.2019.01010 (PMC6514248; doi:10.3389/fmicb.2019.01010)
Supplement: Supplementary file 1 [file Table_1.DOCX]

Supplementary Table S1 Strains used in this study

| Strain | Description | Reference |
| --- | --- | --- |
| BW25113 (wild type) | *F*^-^, *Δ(araD-araB)567 Δ(rhaD-rhaB)568 ΔlacZ4787 (::rrnB-3) hsdR514 rph-1* | Grenier et al., 2014 |
| *gltA*+ | AG1(ME5305) *recA1 endA1 gyrA96 thi-1* *hs*dR17(r_K_^-^/m_K_^+^) *supE44 relA1* with a pCA24N plasmid carrying the *gltA* | Kitagawa et al., 2005 |
| Δ*pta* | BW25113 Δ*pta*::*tetA ** | Baba et al., 2006 |

* The original kanamycin resistance gene was changed to tetracycline resistance gene for a reason not related to this work.

Grenier, F., Matteau, D., Baby, V., Rodrigue, S. (2014). Complete Genome Sequence of *Escherichia coli* BW25113. Genome Announc. 2, e01038-14.

Kitagawa, M., Ara, T., Arifuzzaman, M., Ioka-Nakamichi, T., Inamoto, E., Toyonaga, H., and Mori, H. (2005). Complete set of ORF clones of *Escherichia coli* ASKA library (a complete set of *E. coli* K-12 ORF archive): unique resources for biological research. DNA Res. 12, 291-299.

Baba, T., Ara, T., Hasegawa, M., Takai, Y., Okumura, Y., Baba, M., Datsenko, K.A., Tomita, M., Wanner, B.L., Mori, H. (2006). Construction of Escherichia coli K-12 in-frame, single-gene knockout mutants: the Keio collection. Mol. Syst. Biol. 2, 2006.0008.
