# Supplementary material for: 13C-Metabolic Flux Analysis Reveals Effect of Phenol on Central Carbon Metabolism in Escherichia coli
Source: Front Microbiol. 2019 May 7;10:1010. doi: 10.3389/fmicb.2019.01010 (PMC6514248; doi:10.3389/fmicb.2019.01010)
Supplement: Supplementary file 2 [file Table_2.DOCX]

Supplementary Table S2 Metabolic model

| Reaction number | Reaction | Carbon transition |
| --- | --- | --- |
| **Glycolysis** | |  |
| r1 | Subs_Glc --> G6P | ABCDEF --> ABCDEF |
| r2 | G6P --> F6P | ABCDEF --> ABCDEF |
| r3 | F6P --> G6P | ABCDEF --> ABCDEF |
| r4 | F6P --> FBP | ABCDEF --> ABCDEF |
| r5 | FBP --> DHAP + G3P | ABCDEF --> CBA + DEF |
| r6 | DHAP + G3P --> FBP | CBA + DEF --> ABCDEF |
| r7 | DHAP --> G3P | ABC --> ABC |
| r8 | G3P --> DHAP | ABC --> ABC |
| r9 | G3P --> PGP | ABC --> ABC |
| r10 | PGP --> G3P | ABC --> ABC |
| r11 | PGP --> PEP | ABC --> ABC |
| r12 | PEP --> PGP | ABC --> ABC |
| r13 | PEP --> PYR | ABC --> ABC |
| **TCA cycle** | |  |
| r14 | PYR --> AcCoA + CO2_in | ABC --> BC + A |
| r15 | AcCoA + OAA --> IsoCit | AB + CDEF --> FEDBAC |
| r16 | IsoCit --> AKG + CO2_in | ABCDEF --> ABCDE + F |
| r17 | AKG --> Sym_SUC + CO2_in | ABCDE --> BCDE + A |
| r18 | Sym_SUC --> MAL | ABCD --> ABCD |
| r19 | MAL --> Sym_SUC | ABCD --> ABCD |
| r20 | MAL --> OAA | ABCD --> ABCD |
| r21 | OAA --> MAL | ABCD --> ABCD |
| r22 | IsoCit + AcCoA --> MAL + Sym_SUC | ABCDEF + GH --> ABHG + FCDE |
| **Anaplerosis** | |  |
| r23 | PEP + CO2_in --> OAA | ABC + D --> ABCD |
| r24 | OAA --> PEP + CO2_in | ABCD --> ABC + D |
| r25 | MAL --> PYR + CO2_in | ABCD --> ABC + D |

Table S1 continued

| Reaction number | Reaction | Carbon transition |
| --- | --- | --- |
| **Pentose phosphate pathway** | |  |
| r26 | G6P --> x6PG | ABCDEF --> ABCDEF |
| r27 | x6PG --> Ru5P + CO2_in | ABCDEF --> BCDEF + A |
| r28 | Ru5P --> X5P | ABCDE --> ABCDE |
| r29 | X5P --> Ru5P | ABCDE --> ABCDE |
| r30 | Ru5P --> R5P | ABCDE --> ABCDE |
| r31 | R5P --> Ru5P | ABCDE --> ABCDE |
| r32 | R5P + X5P --> S7P + G3P | ABCDE + FGHIJ --> FGABCDE + HIJ |
| r33 | G3P + S7P --> X5P + R5P | HIJ + FGABCDE --> FGHIJ + ABCDE |
| r34 | G3P + S7P --> F6P + E4P | ABC + DEFGHIJ --> DEFABC + GHIJ |
| r35 | E4P + F6P --> S7P + G3P | GHIJ + DEFABC --> DEFGHIJ + ABC |
| r36 | E4P + X5P --> F6P + G3P | ABCD + EFGHI --> EFABCD + GHI |
| r37 | G3P + F6P --> X5P + E4P | GHI + EFABCD --> EFGHI + ABCD |
| **Entner-Doudoroff pathway** | |  |
| r38 | x6PG --> PYR + G3P | ABCDEF --> ABC + DEF |
| **Amino acid metabolism** | |  |
| r39 | PGP --> Ser | ABC --> ABC |
| r40 | Ser --> Gly +THF_in | ABC --> AB + C |
| r41 | Gly + THF_in --> Ser | AB + C --> ABC |
| **CO_2_ exchange** |  |  |
| r42 | Subs_CO2 --> CO2_in | A --> A |
| r43 | CO2_in --> [CO2_ex] |  |
| r44 | Subs_THF --> THF_in | A --> A |
| r45 | THF_in --> [THF_ex] |  |
| **Acetate production** | |  |
| r46 | AcCoA --> [Acetate] |  |
| **Biomass synthesis** | |  |
| r47 | G6P --> [Biomass] |  |
