# Supplementary material for: 13C-Metabolic Flux Analysis Reveals Effect of Phenol on Central Carbon Metabolism in Escherichia coli
Source: Front Microbiol. 2019 May 7;10:1010. doi: 10.3389/fmicb.2019.01010 (PMC6514248; doi:10.3389/fmicb.2019.01010)
Supplement: Supplementary file 3 [file Table_3.DOCX]

Supplementary Table S3 Measured and estimated MDV of phenol concentration 0%, 0.1%, 0.15% using proteogenic amino acid

| Amino  acid | Fragment | MDV | phenol concentration | | | | | |
| --- | --- | --- | --- | --- | --- | --- | --- | --- |
|  |  |  | 0% | 0% | 0.1% | 0.1% | 0.15% | 0.15% |
|  |  |  | measured | estimated | measured | estimated | measured | estimated |
| Ala | m_57 | M+0 | 0.588 | 0.582 | 0.574 | 0.565 | 0.570 | 0.560 |
|  |  | M+1 | 0.401 | 0.390 | 0.407 | 0.402 | 0.412 | 0.407 |
|  |  | M+2 | 0.011 | 0.027 | 0.018 | 0.032 | 0.018 | 0.031 |
|  |  | M+3 | 0.000 | 0.001 | 0.000 | 0.001 | 0.000 | 0.001 |
|  | m_85 | M+0 | 0.599 | 0.607 | 0.588 | 0.599 | 0.585 | 0.594 |
|  |  | M+1 | 0.396 | 0.380 | 0.404 | 0.385 | 0.407 | 0.389 |
|  |  | M+2 | 0.005 | 0.013 | 0.008 | 0.016 | 0.007 | 0.017 |
| Asx | m_57 | M+0 | 0.471 | 0.459 | 0.489 | 0.483 | 0.501 | 0.493 |
|  |  | M+1 | 0.421 | 0.424 | 0.422 | 0.421 | 0.423 | 0.422 |
|  |  | M+2 | 0.101 | 0.106 | 0.084 | 0.087 | 0.073 | 0.079 |
|  |  | M+3 | 0.007 | 0.010 | 0.005 | 0.008 | 0.003 | 0.006 |
|  |  | M+4 | 0.000 | 0.000 | 0.000 | 0.000 | 0.000 | 0.000 |
|  | m_85 | M+0 | 0.517 | 0.515 | 0.531 | 0.531 | 0.537 | 0.536 |
|  |  | M+1 | 0.414 | 0.411 | 0.414 | 0.408 | 0.418 | 0.409 |
|  |  | M+2 | 0.066 | 0.069 | 0.053 | 0.058 | 0.044 | 0.053 |
|  |  | M+3 | 0.002 | 0.004 | 0.001 | 0.003 | 0.001 | 0.002 |
|  | m_159 | M+0 | 0.512 | 0.515 | 0.526 | 0.531 | 0.531 | 0.536 |
|  |  | M+1 | 0.408 | 0.411 | 0.409 | 0.408 | 0.412 | 0.409 |
|  |  | M+2 | 0.071 | 0.069 | 0.057 | 0.058 | 0.049 | 0.053 |
|  |  | M+3 | 0.010 | 0.004 | 0.009 | 0.003 | 0.008 | 0.002 |
|  | m_302 | M+0 | 0.740 | 0.753 | 0.765 | 0.776 | 0.772 | 0.788 |
|  |  | M+1 | 0.240 | 0.234 | 0.220 | 0.213 | 0.213 | 0.202 |
|  |  | M+2 | 0.019 | 0.014 | 0.016 | 0.011 | 0.015 | 0.010 |

Table S3 continued

| Amino  acid | Fragment | MDV | phenol concentration | | | | | |
| --- | --- | --- | --- | --- | --- | --- | --- | --- |
|  |  |  | 0% | 0% | 0.1% | 0.1% | 0.15% | 0.15% |
|  |  |  | measured | estimated | measured | estimated | measured | estimated |
| Glx | m_57 | M+0 | 0.332 | 0.313 | 0.337 | 0.318 | 0.336 | 0.318 |
|  |  | M+1 | 0.431 | 0.445 | 0.437 | 0.449 | 0.444 | 0.451 |
|  |  | M+2 | 0.203 | 0.205 | 0.198 | 0.201 | 0.196 | 0.200 |
|  |  | M+3 | 0.033 | 0.034 | 0.027 | 0.031 | 0.023 | 0.029 |
|  |  | M+4 | 0.002 | 0.002 | 0.001 | 0.002 | 0.001 | 0.002 |
|  |  | M+5 | 0.000 | 0.000 | 0.000 | 0.000 | 0.000 | 0.000 |
|  | m_85 | M+0 | 0.368 | 0.350 | 0.370 | 0.348 | 0.364 | 0.343 |
|  |  | M+1 | 0.441 | 0.455 | 0.447 | 0.456 | 0.453 | 0.458 |
|  |  | M+2 | 0.176 | 0.176 | 0.173 | 0.177 | 0.174 | 0.180 |
|  |  | M+3 | 0.015 | 0.018 | 0.011 | 0.018 | 0.010 | 0.018 |
|  |  | M+4 | 0.000 | 0.000 | 0.000 | 0.001 | 0.000 | 0.001 |
|  | m_159 | M+0 | 0.369 | 0.350 | 0.370 | 0.348 | 0.365 | 0.343 |
|  |  | M+1 | 0.440 | 0.455 | 0.446 | 0.456 | 0.451 | 0.458 |
|  |  | M+2 | 0.175 | 0.176 | 0.172 | 0.177 | 0.174 | 0.180 |
|  |  | M+3 | 0.016 | 0.018 | 0.012 | 0.018 | 0.010 | 0.018 |
|  |  | M+4 | 0.001 | 0.000 | 0.001 | 0.001 | 0.001 | 0.001 |
| Gly | m_57 | M+0 | 0.962 | 0.966 | 0.962 | 0.963 | 0.959 | 0.959 |
|  |  | M+1 | 0.037 | 0.033 | 0.037 | 0.036 | 0.040 | 0.040 |
|  |  | M+2 | 0.001 | 0.001 | 0.002 | 0.001 | 0.002 | 0.001 |
|  | m_85 | M+0 | 0.981 | 0.981 | 0.982 | 0.982 | 0.979 | 0.981 |
|  |  | M+1 | 0.019 | 0.019 | 0.018 | 0.018 | 0.021 | 0.019 |

Table S3 continued

| Amino  acid | Fragment | MDV | phenol concentration | | | | | |
| --- | --- | --- | --- | --- | --- | --- | --- | --- |
|  |  |  | 0% | 0% | 0.1% | 0.1% | 0.15% | 0.15% |
|  |  |  | measured | estimated | measured | estimated | measured | estimated |
| Ile | m_85 | M+0 | 0.319 | 0.313 | 0.321 | 0.318 | 0.321 | 0.318 |
|  |  | M+1 | 0.432 | 0.445 | 0.437 | 0.449 | 0.441 | 0.451 |
|  |  | M+2 | 0.214 | 0.205 | 0.211 | 0.201 | 0.211 | 0.200 |
|  |  | M+3 | 0.034 | 0.034 | 0.029 | 0.031 | 0.026 | 0.029 |
|  |  | M+4 | 0.002 | 0.002 | 0.001 | 0.002 | 0.001 | 0.002 |
|  |  | M+5 | 0.000 | 0.000 | 0.000 | 0.000 | 0.000 | 0.000 |
|  | m_159 | M+0 | 0.318 | 0.313 | 0.321 | 0.318 | 0.321 | 0.318 |
|  |  | M+1 | 0.429 | 0.445 | 0.435 | 0.449 | 0.439 | 0.451 |
|  |  | M+2 | 0.214 | 0.205 | 0.212 | 0.201 | 0.211 | 0.200 |
|  |  | M+3 | 0.035 | 0.034 | 0.030 | 0.031 | 0.027 | 0.029 |
|  |  | M+4 | 0.003 | 0.002 | 0.002 | 0.002 | 0.002 | 0.002 |
|  |  | M+5 | 0.001 | 0.000 | 0.001 | 0.000 | 0.001 | 0.000 |
| Leu | m_85 | M+0 | 0.256 | 0.239 | 0.259 | 0.235 | 0.253 | 0.230 |
|  |  | M+1 | 0.403 | 0.429 | 0.397 | 0.426 | 0.397 | 0.425 |
|  |  | M+2 | 0.273 | 0.266 | 0.274 | 0.269 | 0.278 | 0.273 |
|  |  | M+3 | 0.066 | 0.063 | 0.068 | 0.066 | 0.069 | 0.068 |
|  |  | M+4 | 0.002 | 0.004 | 0.002 | 0.005 | 0.002 | 0.005 |
|  |  | M+5 | 0.000 | 0.000 | 0.000 | 0.000 | 0.000 | 0.000 |
|  | m_159 | M+0 | 0.248 | 0.239 | 0.250 | 0.235 | 0.242 | 0.230 |
|  |  | M+1 | 0.400 | 0.429 | 0.394 | 0.426 | 0.395 | 0.425 |
|  |  | M+2 | 0.278 | 0.266 | 0.279 | 0.269 | 0.284 | 0.273 |
|  |  | M+3 | 0.071 | 0.063 | 0.073 | 0.066 | 0.075 | 0.068 |
|  |  | M+4 | 0.003 | 0.004 | 0.004 | 0.005 | 0.004 | 0.005 |
|  |  | M+5 | 0.000 | 0.000 | 0.000 | 0.000 | 0.000 | 0.000 |

Table S3 continued

| Amino  acid | Fragment | MDV | phenol concentration | | | | | |
| --- | --- | --- | --- | --- | --- | --- | --- | --- |
|  |  |  | 0% | 0% | 0.1% | 0.1% | 0.15% | 0.15% |
|  |  |  | measured | estimated | measured | estimated | measured | estimated |
| Phe | m_57 | M+0 | 0.283 | 0.270 | 0.274 | 0.259 | 0.268 | 0.252 |
|  |  | M+1 | 0.418 | 0.435 | 0.415 | 0.432 | 0.412 | 0.430 |
|  |  | M+2 | 0.243 | 0.240 | 0.250 | 0.247 | 0.255 | 0.252 |
|  |  | M+3 | 0.052 | 0.051 | 0.057 | 0.056 | 0.060 | 0.060 |
|  |  | M+4 | 0.004 | 0.004 | 0.004 | 0.005 | 0.005 | 0.006 |
|  |  | M+5 | 0.000 | 0.000 | 0.000 | 0.000 | 0.000 | 0.000 |
|  |  | M+6 | 0.000 | 0.000 | 0.000 | 0.000 | 0.000 | 0.000 |
|  |  | M+7 | 0.000 | 0.000 | 0.000 | 0.000 | 0.000 | 0.000 |
|  |  | M+8 | 0.000 | 0.000 | 0.000 | 0.000 | 0.000 | 0.000 |
|  |  | M+9 | 0.000 | 0.000 | 0.000 | 0.000 | 0.000 | 0.000 |
|  | m_85 | M+0 | 0.283 | 0.274 | 0.275 | 0.266 | 0.271 | 0.260 |
|  |  | M+1 | 0.419 | 0.438 | 0.415 | 0.436 | 0.413 | 0.434 |
|  |  | M+2 | 0.243 | 0.237 | 0.250 | 0.243 | 0.255 | 0.247 |
|  |  | M+3 | 0.051 | 0.048 | 0.055 | 0.052 | 0.057 | 0.055 |
|  |  | M+4 | 0.003 | 0.003 | 0.004 | 0.004 | 0.004 | 0.004 |
|  |  | M+5 | 0.000 | 0.000 | 0.000 | 0.000 | 0.000 | 0.000 |
|  |  | M+6 | 0.000 | 0.000 | 0.000 | 0.000 | -0.001 | 0.000 |
|  |  | M+7 | 0.000 | 0.000 | 0.000 | 0.000 | 0.000 | 0.000 |
|  |  | M+8 | 0.000 | 0.000 | 0.000 | 0.000 | 0.000 | 0.000 |
|  | m_159 | M+0 | 0.284 | 0.274 | 0.277 | 0.266 | 0.273 | 0.260 |
|  |  | M+1 | 0.418 | 0.438 | 0.414 | 0.436 | 0.412 | 0.434 |
|  |  | M+2 | 0.242 | 0.237 | 0.248 | 0.243 | 0.253 | 0.247 |
|  |  | M+3 | 0.052 | 0.048 | 0.056 | 0.052 | 0.058 | 0.055 |
|  |  | M+4 | 0.004 | 0.003 | 0.005 | 0.004 | 0.005 | 0.004 |
|  |  | M+5 | 0.000 | 0.000 | 0.000 | 0.000 | -0.001 | 0.000 |
|  |  | M+6 | 0.000 | 0.000 | 0.000 | 0.000 | 0.000 | 0.000 |
|  |  | M+7 | 0.000 | 0.000 | 0.000 | 0.000 | 0.000 | 0.000 |
|  |  | M+8 | 0.000 | 0.000 | 0.000 | 0.000 | 0.000 | 0.000 |
|  | m_302 | M+0 | 0.974 | 0.966 | 0.959 | 0.956 | 0.950 | 0.948 |
|  |  | M+1 | 0.027 | 0.033 | 0.040 | 0.043 | 0.048 | 0.051 |
|  |  | M+2 | 0.000 | 0.001 | 0.001 | 0.001 | 0.002 | 0.001 |

Table S3 continued

| Amino  acid | Fragment | MDV | phenol concentration | | | | | |
| --- | --- | --- | --- | --- | --- | --- | --- | --- |
|  |  |  | 0% | 0% | 0.1% | 0.1% | 0.15% | 0.15% |
|  |  |  | measured | estimated | measured | estimated | measured | estimated |
| Ser | m_57 | M+0 | 0.600 | 0.607 | 0.587 | 0.596 | 0.580 | 0.588 |
|  |  | M+1 | 0.394 | 0.381 | 0.405 | 0.392 | 0.410 | 0.399 |
|  |  | M+2 | 0.007 | 0.012 | 0.008 | 0.011 | 0.010 | 0.013 |
|  |  | M+3 | 0.000 | 0.000 | 0.000 | 0.000 | 0.000 | 0.000 |
|  | m_85 | M+0 | 0.607 | 0.617 | 0.597 | 0.610 | 0.590 | 0.603 |
|  |  | M+1 | 0.389 | 0.377 | 0.399 | 0.385 | 0.406 | 0.391 |
|  |  | M+2 | 0.003 | 0.006 | 0.004 | 0.005 | 0.005 | 0.006 |
|  | m_159 | M+0 | 0.608 | 0.617 | 0.598 | 0.610 | 0.591 | 0.603 |
|  |  | M+1 | 0.391 | 0.377 | 0.400 | 0.385 | 0.406 | 0.391 |
|  |  | M+2 | 0.001 | 0.006 | 0.002 | 0.005 | 0.002 | 0.006 |
|  | m_302 | M+0 | 0.971 | 0.966 | 0.969 | 0.963 | 0.965 | 0.959 |
|  |  | M+1 | 0.026 | 0.033 | 0.028 | 0.036 | 0.033 | 0.040 |
|  |  | M+2 | 0.003 | 0.001 | 0.003 | 0.001 | 0.003 | 0.001 |
| Val | m_57 | M+0 | 0.364 | 0.353 | 0.352 | 0.338 | 0.347 | 0.333 |
|  |  | M+1 | 0.458 | 0.458 | 0.456 | 0.458 | 0.458 | 0.460 |
|  |  | M+2 | 0.172 | 0.172 | 0.182 | 0.183 | 0.185 | 0.187 |
|  |  | M+3 | 0.006 | 0.016 | 0.010 | 0.020 | 0.010 | 0.020 |
|  |  | M+4 | 0.000 | 0.001 | 0.000 | 0.001 | 0.000 | 0.001 |
|  |  | M+5 | 0.000 | 0.000 | 0.000 | 0.000 | 0.000 | 0.000 |
|  | m_85 | M+0 | 0.367 | 0.369 | 0.356 | 0.359 | 0.351 | 0.353 |
|  |  | M+1 | 0.456 | 0.461 | 0.456 | 0.461 | 0.457 | 0.463 |
|  |  | M+2 | 0.172 | 0.160 | 0.181 | 0.168 | 0.184 | 0.171 |
|  |  | M+3 | 0.005 | 0.010 | 0.006 | 0.013 | 0.007 | 0.013 |
|  |  | M+4 | 0.000 | 0.000 | 0.000 | 0.000 | 0.000 | 0.000 |
|  | m_159 | M+0 | 0.362 | 0.369 | 0.352 | 0.359 | 0.347 | 0.353 |
|  |  | M+1 | 0.453 | 0.461 | 0.452 | 0.461 | 0.455 | 0.463 |
|  |  | M+2 | 0.169 | 0.160 | 0.178 | 0.168 | 0.180 | 0.171 |
|  |  | M+3 | 0.015 | 0.010 | 0.017 | 0.013 | 0.016 | 0.013 |
|  |  | M+4 | 0.001 | 0.000 | 0.002 | 0.000 | 0.001 | 0.000 |
|  | m_302 | M+0 | 0.905 | 0.909 | 0.877 | 0.880 | 0.874 | 0.879 |
|  |  | M+1 | 0.077 | 0.087 | 0.102 | 0.115 | 0.106 | 0.116 |
|  |  | M+2 | 0.018 | 0.004 | 0.020 | 0.005 | 0.020 | 0.005 |
