# Supplementary material for: 13C-Metabolic Flux Analysis Reveals Effect of Phenol on Central Carbon Metabolism in Escherichia coli
Source: Front Microbiol. 2019 May 7;10:1010. doi: 10.3389/fmicb.2019.01010 (PMC6514248; doi:10.3389/fmicb.2019.01010)
Supplement: Supplementary file 4 [file Table_4.DOCX]

Supplementary Table S4 The best fitted flux distributions and the 95% confidence intervals

(A)

Condition of phenol concentration; 0%

RSS = 92.1

|  |  | Best fit | | The 95% confidence  interval | |
| --- | --- | --- | --- | --- | --- |
| Reaction  number | Reaction | Net flux | Relative  flux  (%) | Lower limit | Upper limit |
| r1 | Subs_Glc --> G6P | 9.40 | 100.0 | 9.40 | 9.40 |
| r2 | G6P --> F6P | 5.52 | 58.8 | 5.37 | 6.78 |
| r3 | F6P --> G6P | 0.00 | 0.0 | 4.89 | 8.00 |
| r4 | F6P --> FBP | 7.54 | 80.2 | - | - |
| r5 | FBP --> DHAP + G3P | 7.54 | 80.2 | 4.89 | 8.00 |
| r6 | DHAP + G3P --> FBP | 0.00 | 0.0 | - | - |
| r7 | DHAP --> G3P | 7.54 | 80.2 | 4.89 | 8.00 |
| r8 | G3P --> DHAP | 0.00 | 0.0 | - | - |
| r9 | G3P --> PGP | 15.96 | 169.7 | 13.28 | 16.45 |
| r10 | PGP --> G3P | 0.00 | 0.0 | - | - |
| r11 | PGP --> PEP | 15.07 | 160.4 | 12.37 | 15.60 |
| r12 | PEP --> PGP | 0.00 | 0.0 | - | - |
| r13 | PEP --> PYR | 9.97 | 106.1 | 5.68 | 15.65 |
| r14 | PYR --> AcCoA + CO2_in | 11.47 | 122.0 | 11.10 | 16.37 |
| r15 | AcCoA + OAA --> IsoCit | 3.59 | 38.2 | 3.00 | 4.50 |
| r16 | IsoCit --> AKG + CO2_in | 3.44 | 36.6 | 0.90 | 4.50 |
| r17 | AKG --> Sym_SUC + CO2_in | 2.81 | 29.8 | 0.24 | 3.88 |
| r18 | Sym_SUC --> MAL | 2.95 | 31.4 | 2.34 | 3.88 |
| r19 | MAL --> Sym_SUC | 0.00 | 0.0 | - | - |
| r20 | MAL --> OAA | -0.04 | -0.4 | -2.16 | 5.98 |
| r21 | OAA --> MAL | 0.00 | 0.0 | - | - |
| r22 | IsoCit + AcCoA --> MAL + Sym_SUC | 0.15 | 1.6 | 0.00 | 3.60 |
| r23 | PEP + CO2_in --> OAA | 4.68 | 49.8 | -0.47 | 6.25 |
| r24 | OAA --> PEP + CO2_in | 0.00 | 0.0 | - | - |
| r25 | MAL --> PYR + CO2_in | 3.14 | 33.4 | 1.50 | 4.50 |

Table S4A continued

|  |  | Best fit | | The 95% confidence  interval | |
| --- | --- | --- | --- | --- | --- |
| Reaction  number | Reaction | Net flux | Relative  flux  (%) | Lower limit | Upper limit |
| r26 | G6P --> x6PG | 3.76 | 40.0 | 2.50 | 3.90 |
| r27 | x6PG --> Ru5P + CO2_in | 3.72 | 39.6 | 0.00 | 3.90 |
| r28 | Ru5P --> X5P | 2.06 | 21.9 | -0.44 | 2.19 |
| r29 | X5P --> Ru5P | 0.00 | 0.0 | - | - |
| r30 | Ru5P --> R5P | 1.66 | 17.7 | 0.41 | 1.74 |
| r31 | R5P --> Ru5P | 0.00 | 0.0 | - | - |
| r32 | R5P + X5P --> S7P + G3P | 1.13 | 12.1 | -0.11 | 1.20 |
| r33 | G3P + S7P --> X5P + R5P | 0.00 | 0.0 | - | - |
| r34 | G3P + S7P --> F6P + E4P | 1.13 | 12.1 | -0.11 | 1.20 |
| r35 | E4P + F6P --> S7P + G3P | 0.00 | 0.0 | - | - |
| r36 | E4P + X5P --> F6P + G3P | 0.92 | 9.8 | -0.33 | 0.99 |
| r37 | G3P + F6P --> X5P + E4P | 0.00 | 0.0 | - | - |
| r38 | x6PG --> PYR + G3P | 0.03 | 0.4 | 0.00 | 3.90 |
| r39 | PGP --> Ser | 0.88 | 9.4 | 0.85 | 0.91 |
| r40 | Ser --> Gly + THF_in | 0.34 | 3.7 | 0.33 | 0.36 |
| r41 | Gly + THF_in --> Ser | 0.00 | 0.0 | - | - |
| r42 | Subs_CO2 --> CO2_in | 0.00 | 0.0 | - | - |
| r43 | CO2_in --> [CO2_ex] | 19.89 | 211.6 | 15.94 | 22.12 |
| r44 | Subs_THF --> THF_in | -0.34 | -3.7 | - | - |
| r45 | THF_in --> [THF_ex] | 0.00 | 0.0 | - | - |
| r46 | AcCoA --> [Acetate] | 6.00 | 63.9 | 4.76 | 8.10 |
| r47 | G6P --> [Biomass] | 0.59 | 6.3 | 0.57 | 0.61 |

(B)

Condition of phenol concentration; 0.1%

RSS = 104.5

|  |  | Best fit | | | |
| --- | --- | --- | --- | --- | --- |
| Reaction  number | Reaction | Net flux | Relative  flux  (%) | Relative  flux  (%) | (0.1% Relative - 0% Relative)  (%) |
| r1 | Subs_Glc --> G6P | 7.70 | 100.0 | 100.0 | 0.0 |
| r2 | G6P --> F6P | 4.88 | 63.4 | 58.8 | 4.7 |
| r3 | F6P --> G6P | 0.00 | 0.0 | 0.0 | 0.0 |
| r4 | F6P --> FBP | 6.30 | 81.9 | 80.2 | 1.7 |
| r5 | FBP --> DHAP + G3P | 6.30 | 81.9 | 80.2 | 1.7 |
| r6 | DHAP + G3P --> FBP | 0.00 | 0.0 | 0.0 | 0.0 |
| r7 | DHAP --> G3P | 6.30 | 81.9 | 80.2 | 1.7 |
| r8 | G3P --> DHAP | 0.00 | 0.0 | 0.0 | 0.0 |
| r9 | G3P --> PGP | 13.24 | 171.9 | 169.7 | 2.2 |
| r10 | PGP --> G3P | 0.00 | 0.0 | 0.0 | 0.0 |
| r11 | PGP --> PEP | 12.55 | 163.0 | 160.4 | 2.6 |
| r12 | PEP --> PGP | 0.00 | 0.0 | 0.0 | 0.0 |
| r13 | PEP --> PYR | 8.58 | 111.4 | 106.1 | 5.4 |
| r14 | PYR --> AcCoA + CO2_in | 9.87 | 128.1 | 122.0 | 6.1 |
| r15 | AcCoA + OAA --> IsoCit | 0.98 | 12.8 | 38.2 | -25.4 |
| r16 | IsoCit --> AKG + CO2_in | 0.76 | 9.9 | 36.6 | -26.7 |
| r17 | AKG --> Sym_SUC + CO2_in | 0.26 | 3.4 | 29.8 | -26.4 |
| r18 | Sym_SUC --> MAL | 0.49 | 6.3 | 31.4 | -25.1 |
| r19 | MAL --> Sym_SUC | 0.00 | 0.0 | 0.0 | 0.0 |
| r20 | MAL --> OAA | -1.83 | -23.8 | -0.4 | -23.4 |
| r21 | OAA --> MAL | 0.00 | 0.0 | 0.0 | 0.0 |
| r22 | IsoCit + AcCoA --> MAL + Sym_SUC | 0.22 | 2.9 | 1.6 | 1.3 |
| r23 | PEP + CO2_in --> OAA | 3.64 | 47.3 | 49.8 | -2.5 |
| r24 | OAA --> PEP + CO2_in | 0.00 | 0.0 | 0.0 | 0.0 |
| r25 | MAL --> PYR + CO2_in | 2.54 | 33.0 | 33.4 | -0.3 |

Table S4B continued

|  |  | Best fit | | | |
| --- | --- | --- | --- | --- | --- |
| Reaction  number | Reaction | Net flux | Relative  flux  (%) | Relative  flux  (%) | (0.1% Relative - 0% Relative)  (%) |
| r26 | G6P --> x6PG | 2.72 | 35.3 | 40.0 | -4.6 |
| r27 | x6PG --> Ru5P + CO2_in | 2.68 | 34.8 | 39.6 | -4.8 |
| r28 | Ru5P --> X5P | 1.45 | 18.9 | 21.9 | -3.0 |
| r29 | X5P --> Ru5P | 0.00 | 0.0 | 0.0 | 0.0 |
| r30 | Ru5P --> R5P | 1.22 | 15.9 | 17.7 | -1.8 |
| r31 | R5P --> Ru5P | 0.00 | 0.0 | 0.0 | 0.0 |
| r32 | R5P + X5P --> S7P + G3P | 0.81 | 10.5 | 12.1 | -1.6 |
| r33 | G3P + S7P --> X5P + R5P | 0.00 | 0.0 | 0.0 | 0.0 |
| r34 | G3P + S7P --> F6P + E4P | 0.81 | 10.5 | 12.1 | -1.6 |
| r35 | E4P + F6P --> S7P + G3P | 0.00 | 0.0 | 0.0 | 0.0 |
| r36 | E4P + X5P --> F6P + G3P | 0.64 | 8.4 | 9.8 | -1.4 |
| r37 | G3P + F6P --> X5P + E4P | 0.00 | 0.0 | 0.0 | 0.0 |
| r38 | x6PG --> PYR + G3P | 0.05 | 0.6 | 0.4 | 0.2 |
| r39 | PGP --> Ser | 0.69 | 8.9 | 9.4 | -0.5 |
| r40 | Ser --> Gly + THF_in | 0.27 | 3.5 | 3.7 | -0.2 |
| r41 | Gly + THF_in --> Ser | 0.00 | 0.0 | 0.0 | 0.0 |
| r42 | Subs_CO2 --> CO2_in | 0.00 | 0.0 | 0.0 | 0.0 |
| r43 | CO2_in --> [CO2_ex] | 12.47 | 162.0 | 211.6 | -49.7 |
| r44 | Subs_THF --> THF_in | -0.27 | -3.5 | -3.7 | 0.2 |
| r45 | THF_in --> [THF_ex] | 0.00 | 0.0 | 0.0 | 0.0 |
| r46 | AcCoA --> [Acetate] | 7.31 | 95.0 | 63.9 | 31.1 |
| r47 | G6P --> [Biomass] | 0.46 | 6.0 | 6.3 | -0.3 |

Table S4B continued

|  |  | The 95% confidence interval | |
| --- | --- | --- | --- |
| Reaction  number | Reaction | Lower limit | Upper limit |
| r1 | Subs_Glc --> G6P | 7.70 | 7.70 |
| r2 | G6P --> F6P | 4.70 | 5.71 |
| r3 | F6P --> G6P | 4.32 | 6.63 |
| r4 | F6P --> FBP | - | - |
| r5 | FBP --> DHAP + G3P | 4.32 | 6.63 |
| r6 | DHAP + G3P --> FBP | - | - |
| r7 | DHAP --> G3P | 4.32 | 6.63 |
| r8 | G3P --> DHAP | - | - |
| r9 | G3P --> PGP | 11.22 | 13.60 |
| r10 | PGP --> G3P | - | - |
| r11 | PGP --> PEP | 10.51 | 12.94 |
| r12 | PEP --> PGP | - | - |
| r13 | PEP --> PYR | 3.18 | 10.16 |
| r14 | PYR --> AcCoA + CO2_in | 9.60 | 10.80 |
| r15 | AcCoA + OAA --> IsoCit | 0.50 | 2.90 |
| r16 | IsoCit --> AKG + CO2_in | 0.50 | 2.20 |
| r17 | AKG --> Sym_SUC + CO2_in | 0.00 | 1.73 |
| r18 | Sym_SUC --> MAL | 0.00 | 2.43 |
| r19 | MAL --> Sym_SUC | - | - |
| r20 | MAL --> OAA | -5.60 | 1.82 |
| r21 | OAA --> MAL | - | - |
| r22 | IsoCit + AcCoA --> MAL + Sym_SUC | 0.00 | 1.44 |
| r23 | PEP + CO2_in --> OAA | 1.93 | 6.98 |
| r24 | OAA --> PEP + CO2_in | - | - |
| r25 | MAL --> PYR + CO2_in | 2.00 | 5.60 |

Table S4B continued

|  |  | The 95% confidence interval | |
| --- | --- | --- | --- |
| Reaction  number | Reaction | Lower limit | Upper limit |
| r26 | G6P --> x6PG | 1.90 | 2.90 |
| r27 | x6PG --> Ru5P + CO2_in | 0.00 | 2.90 |
| r28 | Ru5P --> X5P | -0.35 | 1.62 |
| r29 | X5P --> Ru5P | - | - |
| r30 | Ru5P --> R5P | 0.32 | 1.31 |
| r31 | R5P --> Ru5P | - | - |
| r32 | R5P + X5P --> S7P + G3P | -0.09 | 0.89 |
| r33 | G3P + S7P --> X5P + R5P | - | - |
| r34 | G3P + S7P --> F6P + E4P | -0.09 | 0.89 |
| r35 | E4P + F6P --> S7P + G3P | - | - |
| r36 | E4P + X5P --> F6P + G3P | -0.26 | 0.73 |
| r37 | G3P + F6P --> X5P + E4P | - | - |
| r38 | x6PG --> PYR + G3P | 0.00 | 2.90 |
| r39 | PGP --> Ser | 0.66 | 0.72 |
| r40 | Ser --> Gly + THF_in | 0.26 | 0.28 |
| r41 | Gly + THF_in --> Ser | - | - |
| r42 | Subs_CO2 --> CO2_in | - | - |
| r43 | CO2_in --> [CO2_ex] | 9.47 | 16.75 |
| r44 | Subs_THF --> THF_in | - | - |
| r45 | THF_in --> [THF_ex] | - | - |
| r46 | AcCoA --> [Acetate] | 5.05 | 8.96 |
| r47 | G6P --> [Biomass] | 0.44 | 0.48 |

(C)

Condition of phenol concentration; 0.15%

RSS = 103.3

|  |  | Best fit | | | |
| --- | --- | --- | --- | --- | --- |
| Reaction  number | Reaction | Net flux | Relative  flux  (%) | Relative  flux  (%) | (0.15% Relative  - 0% Relative)  (%) |
| r1 | Subs_Glc --> G6P | 7.10 | 100.0 | 100.0 | 0.0 |
| r2 | G6P --> F6P | 4.79 | 67.4 | 58.8 | 8.7 |
| r3 | F6P --> G6P | 0.00 | 0.0 | 0.0 | 0.0 |
| r4 | F6P --> FBP | 5.97 | 84.1 | 80.2 | 3.9 |
| r5 | FBP --> DHAP + G3P | 5.97 | 84.1 | 80.2 | 3.9 |
| r6 | DHAP + G3P --> FBP | 0.00 | 0.0 | 0.0 | 0.0 |
| r7 | DHAP --> G3P | 5.97 | 84.1 | 80.2 | 3.9 |
| r8 | G3P --> DHAP | 0.00 | 0.0 | 0.0 | 0.0 |
| r9 | G3P --> PGP | 12.51 | 176.2 | 169.7 | 6.4 |
| r10 | PGP --> G3P | 0.00 | 0.0 | 0.0 | 0.0 |
| r11 | PGP --> PEP | 12.00 | 169.0 | 160.4 | 8.7 |
| r12 | PEP --> PGP | 0.00 | 0.0 | 0.0 | 0.0 |
| r13 | PEP --> PYR | 6.45 | 90.8 | 106.1 | -15.2 |
| r14 | PYR --> AcCoA + CO2_in | 9.90 | 139.4 | 122.0 | 17.4 |
| r15 | AcCoA + OAA --> IsoCit | 0.37 | 5.2 | 38.2 | -33.0 |
| r16 | IsoCit --> AKG + CO2_in | 0.37 | 5.2 | 36.6 | -31.4 |
| r17 | AKG --> Sym_SUC + CO2_in | 0.00 | 0.0 | 29.8 | -29.8 |
| r18 | Sym_SUC --> MAL | 0.01 | 0.1 | 31.4 | -31.4 |
| r19 | MAL --> Sym_SUC | 0.00 | 0.0 | 0.0 | 0.0 |
| r20 | MAL --> OAA | -4.34 | -61.1 | -0.4 | -60.7 |
| r21 | OAA --> MAL | 0.00 | 0.0 | 0.0 | 0.0 |
| r22 | IsoCit + AcCoA --> MAL + Sym_SUC | 0.00 | 0.0 | 1.6 | -1.5 |
| r23 | PEP + CO2_in --> OAA | 5.31 | 74.8 | 49.8 | 25.0 |
| r24 | OAA --> PEP + CO2_in | 0.00 | 0.0 | 0.0 | 0.0 |
| r25 | MAL --> PYR + CO2_in | 4.34 | 61.2 | 33.4 | 27.8 |

Table S4C continued

|  |  | Best fit | | | |
| --- | --- | --- | --- | --- | --- |
| Reaction  number | Reaction | Net flux | Relative  flux  (%) | Relative  flux  (%) | (0.15% Relative - 0% Relative)  (%) |
| r26 | G6P --> x6PG | 2.24 | 31.6 | 40.0 | -8.4 |
| r27 | x6PG --> Ru5P + CO2_in | 2.17 | 30.6 | 39.6 | -9.0 |
| r28 | Ru5P --> X5P | 1.21 | 17.0 | 21.9 | -4.9 |
| r29 | X5P --> Ru5P | 0.00 | 0.0 | 0.0 | 0.0 |
| r30 | Ru5P --> R5P | 0.97 | 13.6 | 17.7 | -4.1 |
| r31 | R5P --> Ru5P | 0.00 | 0.0 | 0.0 | 0.0 |
| r32 | R5P + X5P --> S7P + G3P | 0.66 | 9.4 | 12.1 | -2.7 |
| r33 | G3P + S7P --> X5P + R5P | 0.00 | 0.0 | 0.0 | 0.0 |
| r34 | G3P + S7P --> F6P + E4P | 0.66 | 9.4 | 12.1 | -2.7 |
| r35 | E4P + F6P --> S7P + G3P | 0.00 | 0.0 | 0.0 | 0.0 |
| r36 | E4P + X5P --> F6P + G3P | 0.54 | 7.6 | 9.8 | -2.2 |
| r37 | G3P + F6P --> X5P + E4P | 0.00 | 0.0 | 0.0 | 0.0 |
| r38 | x6PG --> PYR + G3P | 0.07 | 1.0 | 0.4 | 0.6 |
| r39 | PGP --> Ser | 0.51 | 7.1 | 9.4 | -2.3 |
| r40 | Ser --> Gly + THF_in | 0.20 | 2.8 | 3.7 | -0.9 |
| r41 | Gly + THF_in --> Ser | 0.00 | 0.0 | 0.0 | 0.0 |
| r42 | Subs_CO2 --> CO2_in | 0.00 | 0.0 | 0.0 | 0.0 |
| r43 | CO2_in --> [CO2_ex] | 11.48 | 161.6 | 211.6 | -50.0 |
| r44 | Subs_THF --> THF_in | -0.20 | -2.8 | -3.7 | 0.9 |
| r45 | THF_in --> [THF_ex] | 0.00 | 0.0 | 0.0 | 0.0 |
| r46 | AcCoA --> [Acetate] | 8.54 | 120.2 | 63.9 | 56.4 |
| r47 | G6P --> [Biomass] | 0.34 | 4.8 | 6.3 | -1.5 |

Table S4C continued

|  |  | The 95% confidence interval | |
| --- | --- | --- | --- |
| Reaction  number | Reaction | Lower limit | Upper limit |
| r1 | Subs_Glc --> G6P | 7.10 | 7.10 |
| r2 | G6P --> F6P | 4.73 | 5.14 |
| r3 | F6P --> G6P | 4.45 | 6.17 |
| r4 | F6P --> FBP | - | - |
| r5 | FBP --> DHAP + G3P | 4.45 | 6.17 |
| r6 | DHAP + G3P --> FBP | - | - |
| r7 | DHAP --> G3P | 4.45 | 6.17 |
| r8 | G3P --> DHAP | - | - |
| r9 | G3P --> PGP | 10.97 | 12.77 |
| r10 | PGP --> G3P | - | - |
| r11 | PGP --> PEP | 10.45 | 12.32 |
| r12 | PEP --> PGP | - | - |
| r13 | PEP --> PYR | 3.39 | 10.29 |
| r14 | PYR --> AcCoA + CO2_in | 9.73 | 10.90 |
| r15 | AcCoA + OAA --> IsoCit | 0.37 | 1.80 |
| r16 | IsoCit --> AKG + CO2_in | 0.35 | 0.90 |
| r17 | AKG --> Sym_SUC + CO2_in | 0.00 | 0.58 |
| r18 | Sym_SUC --> MAL | 0.00 | 1.45 |
| r19 | MAL --> Sym_SUC | - | - |
| r20 | MAL --> OAA | -5.80 | 0.99 |
| r21 | OAA --> MAL | - | - |
| r22 | IsoCit + AcCoA --> MAL + Sym_SUC | 0.00 | 1.17 |
| r23 | PEP + CO2_in --> OAA | 1.44 | 6.80 |
| r24 | OAA --> PEP + CO2_in | - | - |
| r25 | MAL --> PYR + CO2_in | 1.60 | 5.80 |

Table S4C continued

|  |  | The 95% confidence interval | |
| --- | --- | --- | --- |
| Reaction  number | Reaction | Lower limit | Upper limit |
| r26 | G6P --> x6PG | 1.90 | 2.30 |
| r27 | x6PG --> Ru5P + CO2_in | 0.00 | 2.30 |
| r28 | Ru5P --> X5P | -0.25 | 1.32 |
| r29 | X5P --> Ru5P | - | - |
| r30 | Ru5P --> R5P | 0.22 | 1.02 |
| r31 | R5P --> Ru5P | - | - |
| r32 | R5P + X5P --> S7P + G3P | -0.06 | 0.71 |
| r33 | G3P + S7P --> X5P + R5P | - | - |
| r34 | G3P + S7P --> F6P + E4P | -0.06 | 0.71 |
| r35 | E4P + F6P --> S7P + G3P | - | - |
| r36 | E4P + X5P --> F6P + G3P | -0.19 | 0.60 |
| r37 | G3P + F6P --> X5P + E4P | - | - |
| r38 | x6PG --> PYR + G3P | 0.00 | 2.30 |
| r39 | PGP --> Ser | 0.45 | 0.52 |
| r40 | Ser --> Gly + THF_in | 0.17 | 0.20 |
| r41 | Gly + THF_in --> Ser | - | - |
| r42 | Subs_CO2 --> CO2_in | - | - |
| r43 | CO2_in --> [CO2_ex] | 9.87 | 14.62 |
| r44 | Subs_THF --> THF_in | - | - |
| r45 | THF_in --> [THF_ex] | - | - |
| r46 | AcCoA --> [Acetate] | 6.91 | 9.65 |
| r47 | G6P --> [Biomass] | 0.30 | 0.35 |
