# Supplementary material for: 13C-Metabolic Flux Analysis Reveals Effect of Phenol on Central Carbon Metabolism in Escherichia coli
Source: Front Microbiol. 2019 May 7;10:1010. doi: 10.3389/fmicb.2019.01010 (PMC6514248; doi:10.3389/fmicb.2019.01010)
Supplement: Supplementary file 5 [file Table_5.DOCX]

**Supplementary Figure 1**

**Relationship between phenol concentration and cell growth of wild type (WT) and citrate synthase overexpressed (*gltA*+) strains.**

Closed circles and squares represent the maximum specific growth rate (h^-1^) and the maximum OD_660_ of wild type during the 24 h period. Orange close circles and squares represent of citrate synthase overexpressed strain (*gltA*+).
